# Telemedicine Rapid Assessment in the Emergency Department: A Pilot Study

**DOI:** 10.1089/tmr.2021.0029

**Published:** 2021-11-19

**Authors:** Devin Peuser, Paul Mangasarian, Jorge Otero, Tamara Scott, Joshua W. Elder

**Affiliations:** ^1^ Department of Emergency Medicine, Mohawk Valley Health System, Utica, New York, USA.; ^2^ Department of Emergency Medicine, School of Medicine, University of California Davis, Sacramento, California, USA.; ^3^ Department of Medicine, Yale University School of Medicine, New Haven, Connecticut, USA.; ^4^ Ambulatory Practice Innovation, University of California Davis Health, Sacramento, California, USA.

**Keywords:** telehealth, rapid assessment, emergency department, telemedicine

## Abstract

**Background:** The objective of this study is to evaluate a rapid assessment telemedicine system in the emergency department for safety, workflow disruption, and satisfaction level by clinicians, staff, and patients.

**Methods:** After consent is obtained, the patient and telemedicine emergency physician are connected on a Health Insurance Portability and Accountability Act-compliant video conference for a rapid assessment, including history of present illness, review of systems, and past medical history. The telemedicine physician initiates orders based on these data. Patient data collected include age, gender, chief complaint, Emergency Severity Index, door to telemedicine physician time, door to in-person physician time, length of stay, left without being seen (LWBS) status, and satisfaction scores. Nurses were assessed for workflow interruptions and communication gaps. In-person physicians were assessed regarding perceived missed information in patient management, perceived workflow interruptions, and satisfaction.

**Results:** Convenience sampling when participating physicians were on-shift resulted in 22 patients enrolled out of 25 approached over 2 months. No patients LWBS, and no in-person physicians perceived missed information in management. Satisfaction was well scored in all evaluated parties, with no score below a 4.77 out of 5 for any of the categories evaluated. Only one case interrupted the workflow of the on-site physician.

**Discussion:** Telemedicine rapid assessment was found to be safe, efficient, and associated with high satisfaction. These results warrant study on a larger scale with a control group to statistically assess differences in outcome metrics such as LWBS rates. If effective, this approach could provide a novel and flexible physician-staffing tool.

## Introduction

Utilizing telemedicine technology in the emergency department (ED) has the potential to increase access to care, as well as timeliness and quality of care. It can build flexibility into physician staffing, adjusting in real time to fluctuating patient volume. These benefits show promise for improving both safety and efficiency for patients and providers alike. However, implementing telemedicine might also lead to workflow disturbances and/or decreased satisfaction for patients or providers. Telemedicine is in need of evaluation across all these metrics. The Agency for Healthcare Research and Quality points to several areas, most notably urgent care, which are in desperate need of more primary literature to support the value of telemedicine.^1^

The unpredictable nature of the ED patient volumes, coupled with tightening hospital budgets, present challenging staffing decisions for hospital administration. Numerous studies have identified longer wait times as a major factor for lower patient satisfaction in the ED.^2^

In addition, one study identified both door to first provider and length of stay (LOS) times as being strongly associated with the percentage of patients leaving before treatment is completed—a major indicator of ED performance^3^ and a critical safety issue as well. Aside from the patient-centric importance, patient satisfaction scores also significantly impact hospital reimbursements. A telemedicine rapid assessment during surges would help to address these concerns efficiently by only employing an additional physician when necessary.

Rapid assessment is a commonly used practice in emergency medicine. In the form of a triage physician, it has proven effective in improving door to doctor times and LOS.^4^ Videoconference telemedicine has yet to be fully evaluated as a tool to perform rapid assessment in place of an in-person physician for patients who are already in a room. A 2018 study^5^ found that in rural EDs, telemedicine capability decreased door-to-provider time and among transferred patients, ED LOS. A 2020 study^6^ assessed telemedicine evaluations for low acuity patients in the ED, finding that 72 h ED returns and patient satisfaction scores were no different for telemedicine versus in-person patients.

This study will evaluate patient safety through a different metric: the rate at which patients leave the ED without being treated. It will additionally survey satisfaction by patients as well as physicians and nurses. We seek to show in this study that a rapid assessment telemedicine system will be safe, will result in high levels of satisfaction by clinicians, staff and patients alike, and will not be perceived as interfering with practice patterns of providers.

This study aims to explore the benefits and drawbacks to providers and patients from a telemedicine-enabled rapid assessment and initial workup in the ED. The data obtained and conclusions drawn from this study may aid and inform future studies, and contribute to the evolution of ED standard of care. By assessing satisfaction of patients, physicians, and staff, we evaluate for broad acceptance in the ED. Measuring time points such as door to telemedicine physician and door to in-person physician, as well as an analysis of patients leaving without being seen will demonstrate program efficacy and safety.

## Methods

### Study design and setting

A prospective study design with convenience sampling was used. Patients were enrolled in a single ED at an urban community hospital, and Level II trauma center in Waterbury, CT with ∼55,000 visits per year.

### Participants

Patients were enrolled in this study only when participating physicians were on-shift, which occurred with no set pattern by time of day or day of week. With a participating physician on-shift, patients presenting for emergency care at Waterbury Hospital and meeting inclusion criteria were approached after triage for enrollment in this study. Research staff then obtain informed consent. Inclusion criteria are as follows:

- Age ≥18 years
- English speaking
- Patient felt able to communicate effectively with an English-speaking physician through the telemedicine system.
- Unseen by providers
- Low acuity (Emergency Severity Index [ESI] 3, 4, 5) automatically, OR higher acuity (ESI 1, 2) when there was no concern for patient safety per the discretion of the participating physician.

Exclusion criteria are as follows:

- Age <18 years
- Non-English speaking or unable to communicate effectively through the telemedicine system
- High acuity (ESI 1, 2), if participating physician expressed safety concerns.

### Intervention

Patients were checked in through standard ED registration process, then seen by a triage nurse, and assigned a room and an appropriate ESI level. Once selected for the study, they were then approached, given a description of the study, and informed consent was obtained. For patients agreeing to participate, a rapid telemedicine assessment was initiated through VSee Clinic™ (VSee, Sunnyvale, CA) with an iPad™. Two emergency physicians staffing the ED on-site worked together to split the patient evaluation: one would see the patient through telemedicine, either from their personal workstation or cell phone equipped with a headset. This physician had no in-person contact with the patient.

During the telemedicine evaluation, an initial history was taken, and laboratories were ordered based on information obtained. The care of the patient was then transferred to the second physician for in-person evaluation and continuation of care. Six different emergency physicians participated as either tele-physicians or in-person physicians. They acted in either role, and in different combinations with each other depending upon staffing that day.

### Methods and measurements

**FIG. 1.**
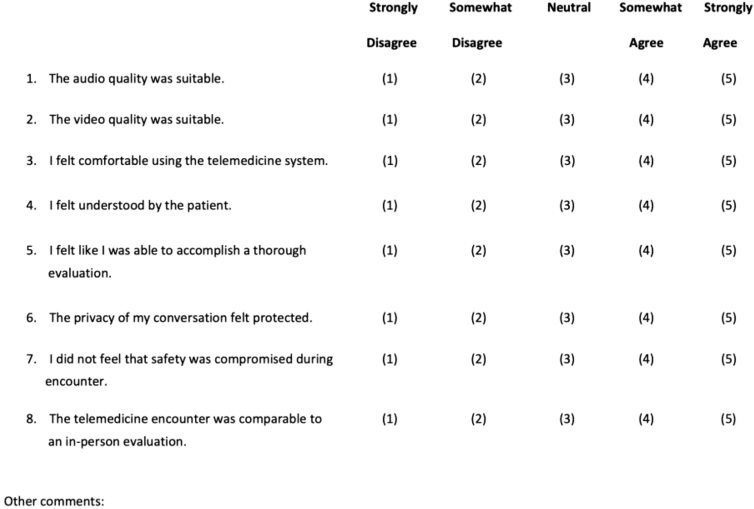
Telemedicine physician satisfaction survey.

**FIG. 2.**
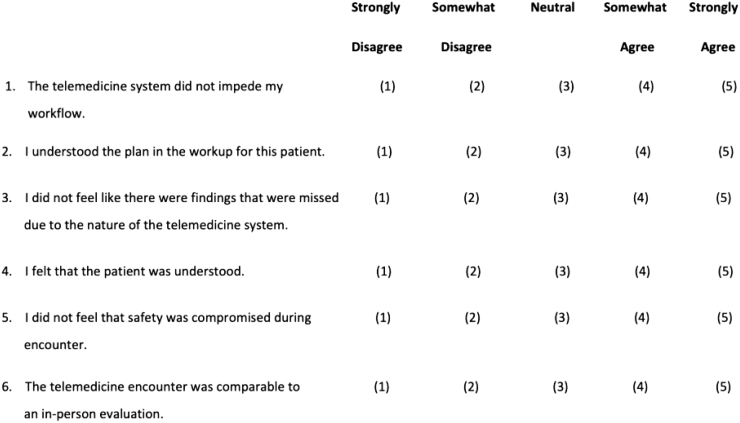
In-person physician satisfaction survey.

**FIG. 3.**
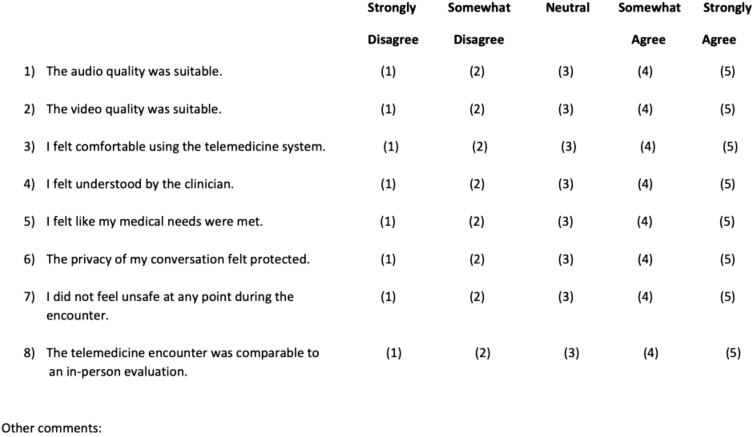
Patient satisfaction survey.

Safety and efficiency outcomes were asked verbally of the in-person physician and nurse immediately after the encounter. Satisfaction was assessed for the patient, the physician performing the telemedicine evaluation, and the physician receiving the patient, through survey handouts completed after the encounter (Figs. 1–3).

Using the Electronic Medical Record (EMR), research staff identified patient demographic and ED visit data, which included age, gender, ESI, and chief complaint. For time measurements, ED arrival (door) time and checkout time were sourced from the EMR, while the telemedicine and in-person physicians recorded their examination start times themselves. Patients were also identified if they left without being seen (LWBS).

### Outcomes

The telemedicine system was assessed across multiple outcome metrics. Primary safety outcomes were perceived missed information on behalf of the in-person providers and patient LWBS status. Time-related outcomes included door to telemedicine physician time, door to in-person physician time, and LOS. Additional outcomes included workflow interruptions as reported by nurses and in-person physicians, as well as patient and physician satisfaction ranked on a Likert scale using a standardized set of questions.

### Analysis

Results were recorded and tabulated in a password-encrypted Microsoft Excel™ spreadsheet. All outcomes were calculated after completion of the study.

This research was approved by the Institutional Review Board at Waterbury Hospital.

## Results

### Characteristics of study subjects

Over a 2-month time period, 22 patients were enrolled out of 25 approached for consent. The mean age was 45 and ranged from 23 to 82 years. Fourteen females and eight males were included in the study. Acuity based on ESI ranged from 2 to 4 (on a 1–5 scale). Chief complaints ranged widely, including chest pain, respiratory, neurological, musculoskeletal, and gastrointestinal complaints. Full patient characteristics are available in Table 1.

**Table 1.** Patient Characteristics

	*n* (%)
Age (years)	
18–30	5 (22.7)
30–50	10 (45.5)
50–65	4 (18.2)
>65	3 (13.6)
Gender	
Male	8 (36.4)
Female	14 (63.6)
ESI	
2	2 (9.1)
3	15 (68.2)
4	5 (22.7)
5	0 (0)
Chief complaint	
Chest pain	4 (18.2)
Respiratory symptoms	1 (4.5)
Neurological	3 (13.6)
MSK	4 (18.2)
Fall	2 (9.1)
GI symptoms	8 (36.4)
Total	22

GI, gastrointestinal; ESI, Emergency Severity Index; MSK, musculoskeletal.

### Main results

Regarding primary safety outcomes, there were zero patients that LWBS and zero cases in which the in-person physician perceived missed information in the telemedicine workup of the patient. Median door to telemedicine doctor time and door to in-person doctor time, in minutes, were 57 (range 14–178) and 187.5 (range 78–383), respectively. Telemedicine physicians started their assessments, on average, 123 min before the on-site physician initiated theirs. Median LOS was 258 min. There were zero cases in which the patient's assigned nurse perceived an interruption in their workflow versus one case in which the on-site physician perceived an interruption.

In this instance, patient volume was low and the on-site physician was able to see the patient in-person before the telemedicine physician. Patient satisfaction scores assessed audio quality (median 5, mean 4.86), video quality (median 5, mean 4.81), medical needs being met (median 5, 4.77), and privacy (median 5, mean 4.77) on a 5-point scale. Tele-physician satisfaction scores addressed audio quality (median 5, mean 5), video quality (median 5, mean 5), telemedicine for evaluation (median 5, mean 5), and safety not being compromised (median 5, mean 5) on the same 5-point scale. Full outcome measures are available in Table 2.

**Table 2.** Primary Outcomes

	Count	
Safety		
Left without being seen		
Yes	0	
No	22	
Missed information in management		
Yes	0	
No	22	
Efficiency		
Interruption of workflow (nurse)		
Yes	0	
No	22	
Interruption of workflow (in-person clinician)		
Yes	1	
No	21	
Plan understood (nurse)		
Yes	22	
No	0	
Plan understood (in-person clinician)		
Yes	21	
No	1	

	Mean (SD)	Median (range)
Patient satisfaction		
The audio quality was suitable	4.86 (0.35)	5 (4–5)
The video quality was suitable	4.77 (0.43)	5 (4–5)
I felt comfortable using the telemedicine	4.77 (0.43)	5 (4–5)
I felt understood by the clinician	4.86 (0.35)	5 (4–5)
I felt like my medical needs were met	4.77 (0.53)	5 (3–5)
The privacy of my conversation felt protected	4.77 (0.43)	5 (4–5)
I did not feel unsafe during any point of the encounter	4.86 (0.35)	5 (4–5)
The telemedicine encounter was comparable with an in-person evaluation	4.55 (0.67)	5 (3–5)
Telemedicine clinician satisfaction		
The audio quality was suitable	5 (0)	5 (5–5)
The video quality was suitable	5 (0)	5 (5–5)
I felt comfortable using the telemedicine system	5 (0)	5 (5–5)
I felt understood by the patient	5 (0)	5 (5–5)
I felt like I was able to accomplish a thorough evaluation	5 (0)	5 (5–5)
The privacy of my conversation felt protected	5 (0)	5 (5–5)
I did not feel that safety was compromised at any point in the encounter	5 (0)	5 (5–5)
The telemedicine encounter was comparable with an in-person evaluation	5 (0)	5 (5–5)
In-person clinician satisfaction		
The telemedicine system did not impede my workflow	5 (0)	5 (5–5)
I understood the plan in the workup for this patient	5 (0)	5 (5–5)
I did not feel like there were findings that were missed due to the nature of the telemedicine system	5 (0)	5 (5–5)
I felt that the patient was understood	5 (0)	5 (5–5)
I did not feel that safety was compromised during the encounter	5 (0)	5 (5–5)
The telemedicine encounter was comparable with an in-person evaluation	5 (0)	5 (5–5)

	Mean (SD)	Median (range)
Time points (minutes)		
Door to telemedicine doctor	71.1 (41.5)	57 (14–178)
Door to in-person doctor	194.5 (78.9)	187.5 (78–383)
Length of stay	283.8 (131.6)	258 (112–633)

SD, standard deviation.

## Discussion

With strong potential to benefit patients, providers, and health care systems as a whole, telemedicine in the ED is growing in both scale and scope. With this growth, robust ongoing evaluation is needed to ensure patient safety, establish best practices, demonstrate value, and improve implementation.

Although small in scale, this study addressed multiple important outcome metrics in its evaluation of telemedicine in ED rapid assessment. Specifically, no patients in the study LWBS, an established marker of patient safety in the ED. Furthermore, there was no perceived missed information by the receiving in-person physician. Our study also found consistently high satisfaction scores for patients and physicians, as well as minimal interruptions to workflow. Overall, this study adds to the primary literature by providing evidence suggesting telemedicine can be well accepted, and implemented safely and efficiently in an ED rapid assessment context.

The in-person physician in this study was asked about workflow interruption both verbally immediately after the encounter, and subsequently on the written satisfaction survey. Notably, in the singular instance in which the in-person physician verbally indicated a workflow interruption, he later marked “strongly agree” on the written survey statement that “the telemedicine system did not impede my workflow.”

Although this might have been recall bias on the part of the physician, it does highlight that a strength of telemedicine in a rapid assessment capacity is its flexibility. In this instance, with low patient volume, the in-person physician was available to see the patient before the telemedicine physician. If the in-person physician would have been forced to wait for an initial telemedicine encounter, this would represent a workflow interruption. The telemedicine system, however, is designed to complement the care of the in-person physician, providing support when needed, and staying out of the way when not needed.

Demand for emergency care is growing. Although the Emergency Medical Treatment and Labor Act is noble in its intentions, it has placed significant strains on hospitals.^7^ Increases in patient volume, boarding, LOS, and LWBS rates affect patients directly as well. Innovative approaches to managing and mitigating these system strains are more important than ever. As such, it is critical to evaluate telemedicine's potential in improving emergency triage.

As the compliance and legal landscape surrounding telemedicine evolves, its continued evaluation is vital to its success. These studies provide a framework to develop national telemedicine policy and strategy, guide initiatives for telemedicine implementation, and generate questions that help refine and identify future areas of use.^8^

The current COVID pandemic has presented a unique opportunity for telemedicine progress. Previous attempts to add ED services to the list of Center for Medicare and Medicaid Services (CMS) criteria for reimbursable telehealth services had failed, citing a lack of evidence supporting the notion that these services are as effective.^9^ However, as a result of the pandemic, CMS temporarily expanded telehealth reimbursements. ED visits are now payable under the Medicare Physician Fee Schedule for the duration of the Public Health Emergency.^10^

Although this signals increasing acceptance of telemedicine in the emergency setting, evidence supporting its efficacy and safety are needed if it is to be utilized long term. Our study demonstrates the utility of telemedicine in ED rapid assessments specifically, an area that has scarce research.

### Limitations

There are important limitations inherent to this study design. Although we were able to demonstrate that the system was well accepted, we did not compare it with a control group. By utilizing a control group, a future study could attempt to compare times (LOS, door to first provider), workups, as well as safety indicators. Another limitation of the design is the use of an in-department physician for the telemedicine component. This was done for this pilot study to facilitate rapid resolution of technical difficulties should they arise. Further research should use an offsite physician, and investigate any concerns with communication or technology functionality. The study size is also relatively small, hindering analysis into whether outcomes differed across patient demographics.

## Conclusion

This pilot study found that telemedicine emergency rapid assessments done by physicians were safe, efficient, and associated with high satisfaction. The results of this study serve as proof of concept for this ED telemedicine initiative. A larger study is being developed to further quantify the effects of this type of protocol. If effective, telemedicine utilizing emergency triage physicians could be a novel and flexible physician-staffing tool with benefits to patients, providers, and the health care system as a whole.

## Abbreviations Used

CMS: Center for Medicare and Medicaid Services
ED: emergency department
ESI: Emergency Severity Index
GI: gastrointestinal
LOS: length of stay
LWBS: left without being seen
MSK: musculoskeletal
SD: standard deviation
